# Supplementary material for: The new normal: Covid-19 risk perceptions and support for continuing restrictions past vaccinations
Source: PLoS One. 2022 Apr 8;17(4):e0266602. doi: 10.1371/journal.pone.0266602 (PMC8993013; doi:10.1371/journal.pone.0266602)
Supplement: S1 Fig — (PDF) [file pone.0266602.s001.pdf]

Supporting information

S1 Fig. Visual Distribution of Core Estimation Indicators (Samples A – D). This section provides additional information that can inform the decision to use the label ‘*over-estimation*’ as opposed to ‘*higher estimation*’.

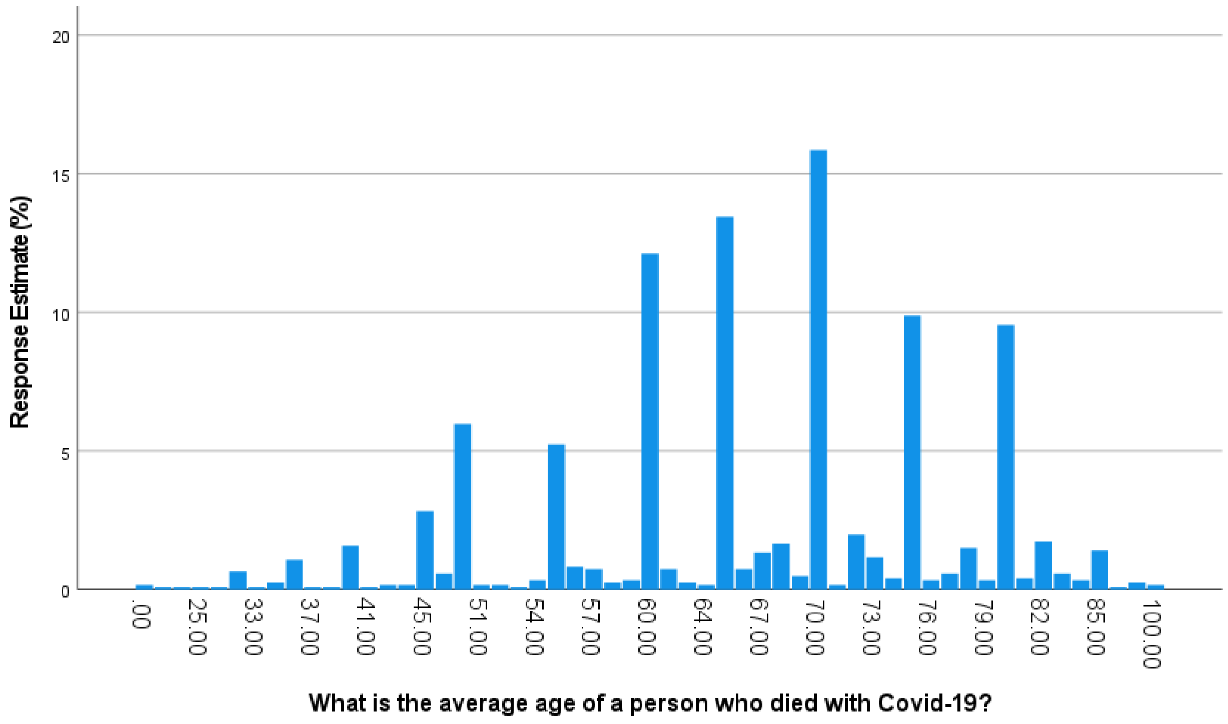

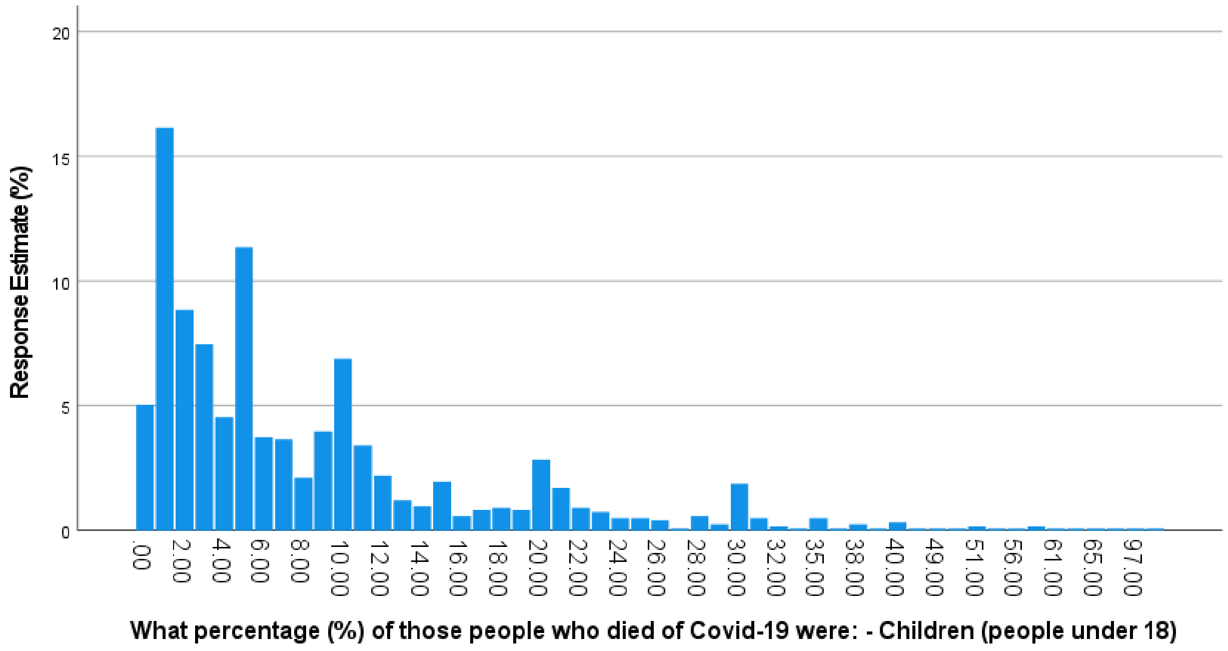
Response Estimate (%)
20
15
10
5
0
.00
2.00
4.00
6.00
8.00
10.00
12.00
14.00
16.00
18.00
20.00
22.00
24.00
26.00
28.00
30.00
32.00
35.00
38.00
40.00
49.00
51.00
56.00
61.00
65.00
97.00
What percentage (%) of those people who died of Covid-19 were: - Children (people under 18)

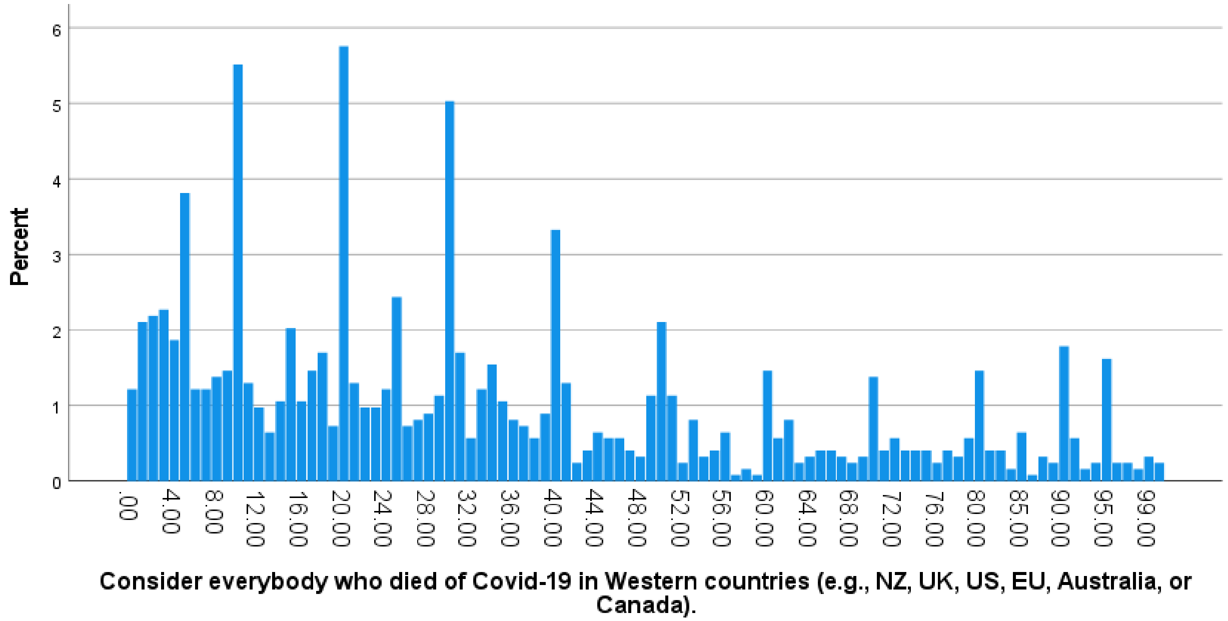

What percentage (%) of those people who died of Covid-19 were: - Healthy people between 18 - 65

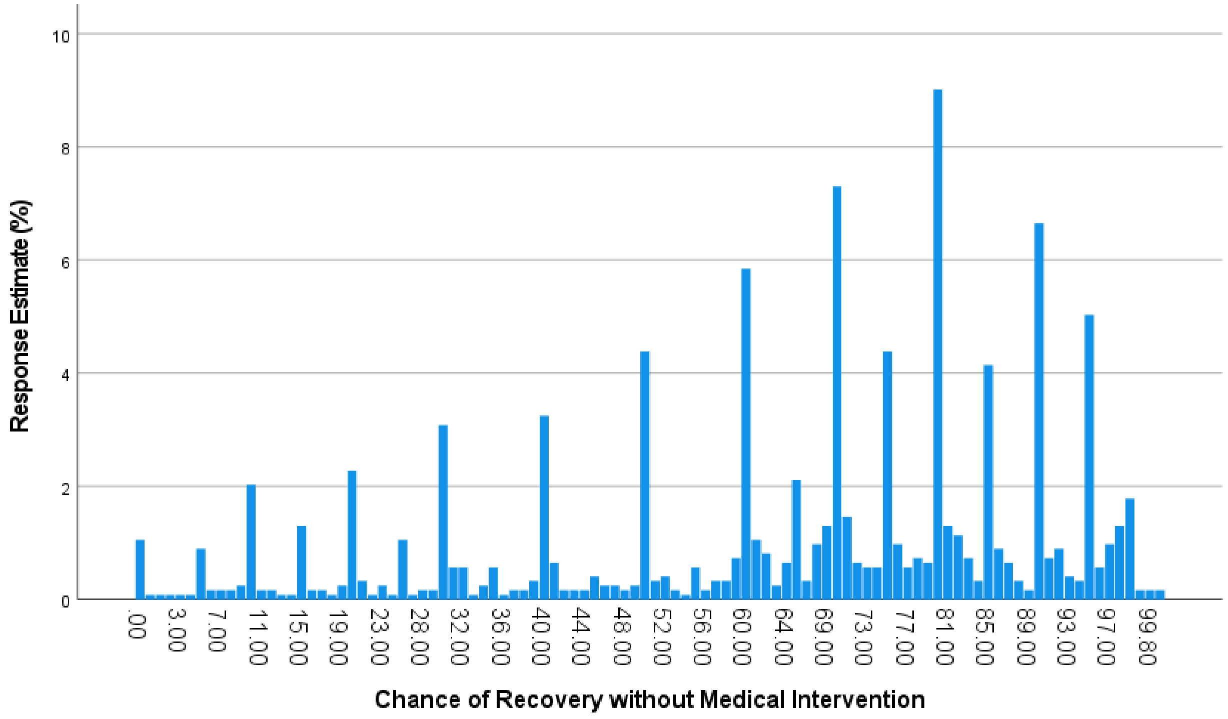
Response Estimate (%)
10
8
6
4
2
0
.00
3.00
7.00
11.00
15.00
19.00
23.00
28.00
32.00
36.00
40.00
44.00
48.00
52.00
56.00
60.00
64.00
69.00
73.00
77.00
81.00
85.00
89.00
93.00
97.00
99.80
Chance of Recovery without Medical Intervention

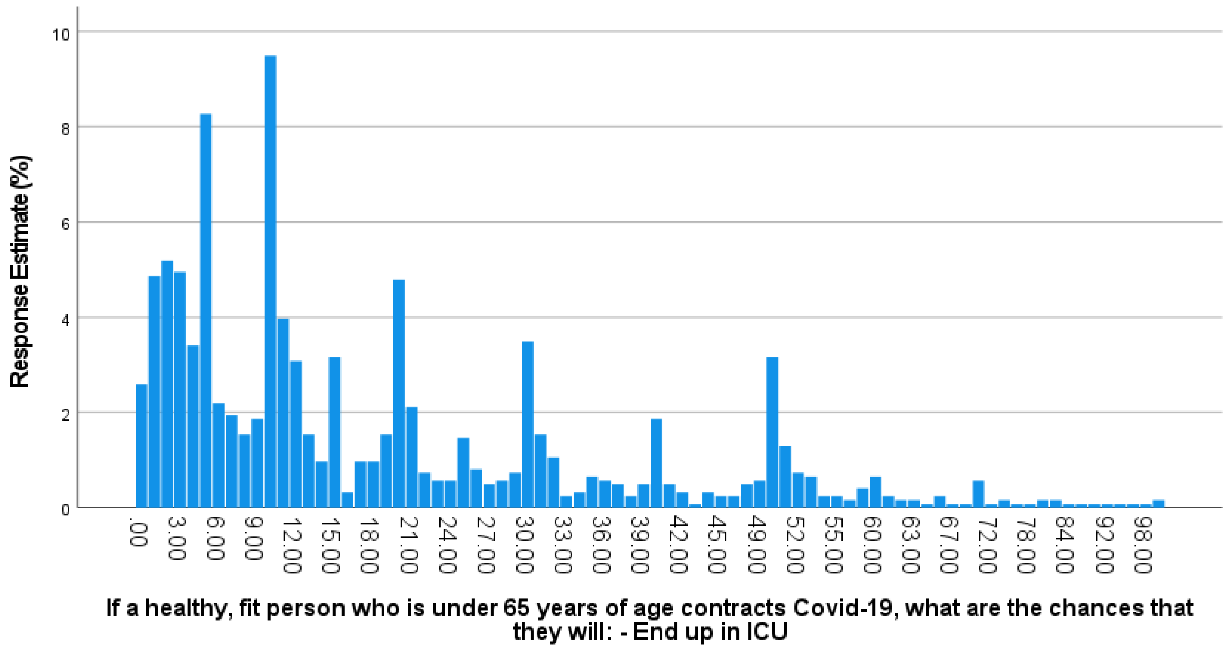
Response Estimate (%)
10
8
6
4
2
0
.00
3.00
6.00
9.00
12.00
15.00
18.00
21.00
24.00
27.00
30.00
33.00
36.00
39.00
42.00
45.00
49.00
52.00
55.00
60.00
63.00
67.00
72.00
78.00
84.00
92.00
98.00
If a healthy, fit person who is under 65 years of age contracts Covid-19, what are the chances that they will: - End up in ICU

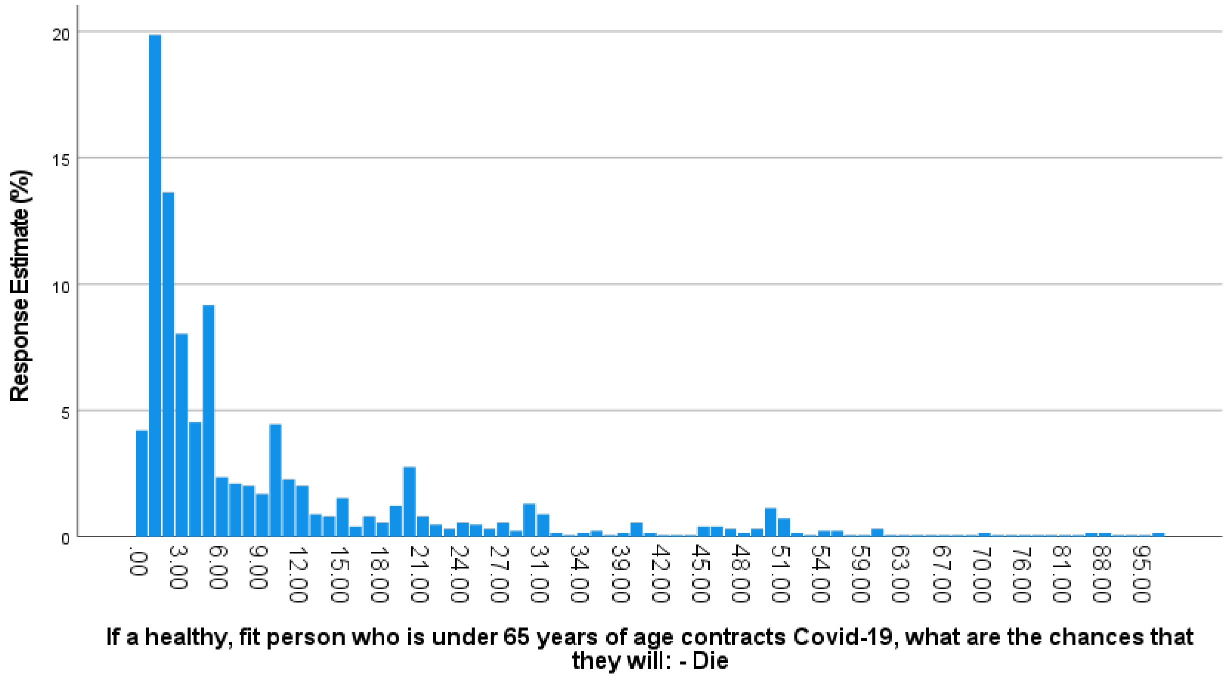

Response Estimate (%)
20
15
10
5
0
.00
3.00
6.00
9.00
12.00
15.00
18.00
21.00
24.00
27.00
31.00
34.00
39.00
42.00
45.00
48.00
51.00
54.00
59.00
63.00
67.00
70.00
76.00
81.00
88.00
95.00
If a healthy, fit person who is under 65 years of age contracts Covid-19, what are the chances that they will: - Die

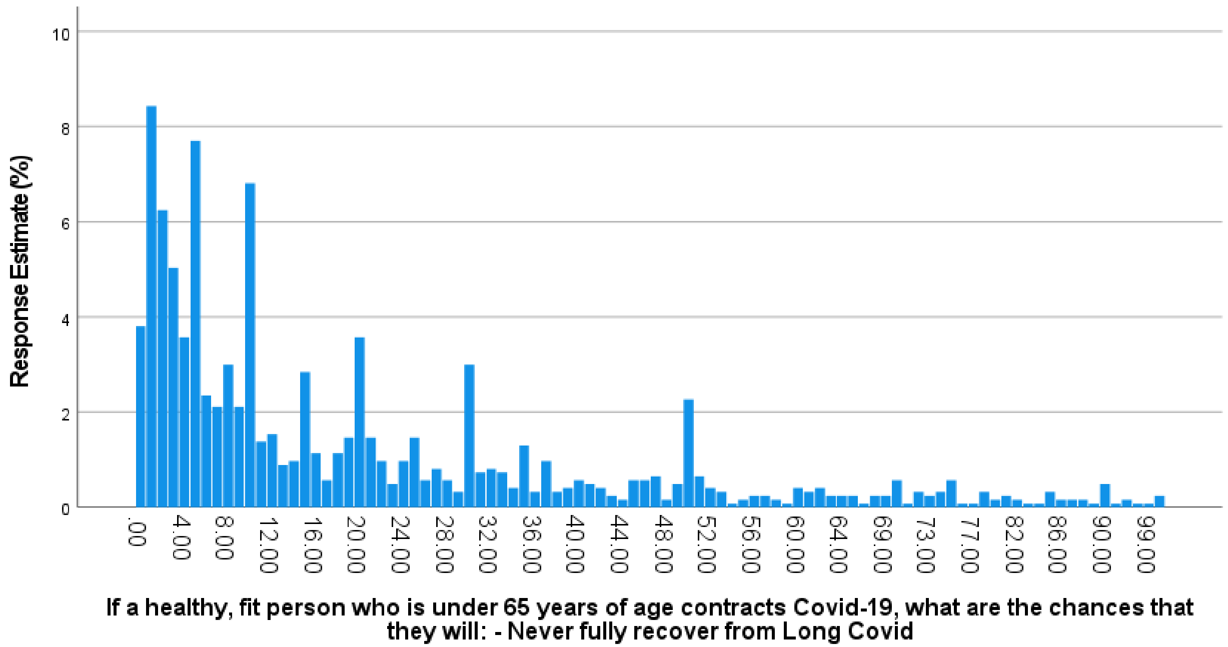

Response Estimate (%)
10
8
6
4
2
0
.00
4.00
8.00
12.00
16.00
20.00
24.00
28.00
32.00
36.00
40.00
44.00
48.00
52.00
56.00
60.00
64.00
69.00
73.00
77.00
82.00
86.00
90.00
99.00
If a healthy, fit person who is under 65 years of age contracts Covid-19, what are the chances that they will: - Never fully recover from Long Covid
