# Supplementary material for: The new normal: Covid-19 risk perceptions and support for continuing restrictions past vaccinations
Source: PLoS One. 2022 Apr 8;17(4):e0266602. doi: 10.1371/journal.pone.0266602 (PMC8993013; doi:10.1371/journal.pone.0266602)
Supplement: S1 Table — (PDF) [file pone.0266602.s002.pdf]

Supporting information

S1 Table: NNP Endorsement by Sample.

		A: Mturk			B: Prolific			C: Prolific			D: ANZ			E: Mturk		
	**Items**	***Mean***	***SD***	***N***	***Mean***	***SD***	***N***	***Mean***	***SD***	***N***	***Mean***	***SD***	***N***	***Mean***	***SD***	***N***
1	If cases are rising, legally require to wear a face mask or covering on all public transport and flights.	**5.71**	1.98	*137*	**6.09**	1.51	*148*	**5.32**	1.90	*253*	**4.73**	2.34	*405*	**5.08**	2.21	*263*
2	Legally require Covid-19 vaccine passport to travel internationally.	**4.59**	2.38	*138*	**4.97**	2.12	*147*	**5.33**	1.93	*251*	**4.80**	2.47	*405*	**4.79**	2.34	*262*
3	Legally require Covid-19 vaccine passports to access institutions (schools, universities, recreation facilities, or workplaces).	**3.95**	2.35	*140*	**4.14**	2.21	*148*	**4.43**	2.06	*251*	**3.80**	2.34	*405*	**4.21**	2.38	*262*
4	If cases are rising, legally require people to wear masks ANY time they are outside (including when they are driving by themselves).	**3.23**	2.35	*140*	**4.16**	2.23	*148*	**4.23**	2.19	*253*	**3.90**	2.30	*404*	**2.97**	2.28	*263*
5	Implement a program similar to COVID-19 alert or levels system to contain flu, which kills thousands of people every year.	**4.88**	1.98	*138*	**4.61**	1.87	*148*	**4.63**	1.84	*251*	**3.39**	2.07	*405*	**4.67**	1.88	*262*
6	Continue strict contact-tracing of all positive Covid-19 cases.	**5.01**	2.14	*140*	**5.55**	1.57	*148*	**5.29**	1.74	*252*	**5.77**	2.12	*402*	**4.61**	2.07	*262*
7	Try to eliminate Covid-19.	**6.20**	1.49	*140*	**6.19**	1.30	*146*	**5.21**	1.78	*252*	**4.93**	2.21	*404*	**4.82**	2.04	*263*
8	Require people who test positive for Covid-19 to self-isolate.	**6.09**	1.61	*139*	**6.19**	1.30	*146*	**6.21**	1.30	*252*	**6.11**	1.79	*401*	**5.74**	1.68	*262*
9	Lift ALL mandates and permit life as normal, even if there are rising cases (Reversed).	**4.89**	2.29	*140*	**5.39**	1.85	*148*	**5.82**	1.60	*252*	**5.50**	2.18	*405*	**4.86**	2.19	*263*
	Cronbach's α		**0.91**			**0.84**			**0.86**			**0.93**			**0.92**	
