# Supplementary material for: The new normal: Covid-19 risk perceptions and support for continuing restrictions past vaccinations
Source: PLoS One. 2022 Apr 8;17(4):e0266602. doi: 10.1371/journal.pone.0266602 (PMC8993013; doi:10.1371/journal.pone.0266602)
Supplement: S2 Table — (PDF) [file pone.0266602.s003.pdf]

Supporting information

S2 Table. Additional Descriptive Information (Samples A – D).

				Skewness		Kurtosis	
Core Variables	*N*	*M*	*SD*	*Statistic*	*SE*	*Statistic*	*SE*
NNP endorsement (9 items)	947	4.98	1.55	**-1.08**	.08	**0.36**	.16
RN Fear (3 items)	280	3.47	1.66	**0.12**	.15	**-1.14**	.29
Average age of C19 death	1204	65.46	12.12	**-0.94**	.07	**1.90**	.14
% of C19 deaths who were children	1233	8.66	10.20	**2.97**	.07	**14.90**	.14
% of C19 deaths - healthy people between 18 - 65	1233	33.68	26.49	**0.82**	.07	**-0.32**	.14
% of people who recover without medical intervention	1232	64.51	25.70	**-0.79**	.07	**-0.32**	.14
% that a healthy person < 65 ends up in ICU	1233	18.78	18.29	**1.45**	.07	**1.98**	.14
% that a healthy person < 65 dies	1233	10.71	15.83	**2.59**	.07	**7.44**	.14
% that a healthy person < 65 never fully recovers from long Covid	1233	19.81	21.80	**1.51**	.07	**1.70**	.14
