# Supplementary material for: The new normal: Covid-19 risk perceptions and support for continuing restrictions past vaccinations
Source: PLoS One. 2022 Apr 8;17(4):e0266602. doi: 10.1371/journal.pone.0266602 (PMC8993013; doi:10.1371/journal.pone.0266602)
Supplement: S3 Table — (PDF) [file pone.0266602.s004.pdf]

Supporting information

ANOVA Results: Presence of Mean Differences Between Samples

The study relies on four distinct samples collected from three sources in 2021, and one source from 2022. The purpose for collecting data from georgaphically distinct sources was to test the generalizability of the positive relationship between the risk estimation and support for the new normal (NNP, RN-fear). Nonetheless, when analyzing the data using all 1,200+ responses for simplicity of presentation, it is crucial to consider differences between the samples. Table S3 only provides comparisons between 2021-based samples, due to their historical similarities.

S3 Table. ANOVA Results.

Variable			*Sum of Squares*	*df*	*Mean Square*	*F*	*p*
1	NNP endorsement	Between Groups	38.34	3	12.781	5.408	0.001
		Within Groups	2228.44	943	2.363		
		Total	2266.78	946			
2	RN: Fear	Between Groups	2.94	1	2.939	1.061	0.304
		Within Groups	770.41	278	2.771		
		Total	773.35	279			
3	Contact-tracing	Between Groups	2366.31	3	788.769	200.037	0.000
		Within Groups	4790.90	1215	3.943		
		Total	7157.20	1218			
4	Compliance	Between Groups	69.36	3	23.120	10.305	0.000
		Within Groups	2723.62	1214	2.244		
		Total	2792.98	1217			
5	Vaccine	Between Groups	12.14	3	4.047	2.390	0.067
		Within Groups	2050.42	1211	1.693		
		Total	2062.56	1214			
6	Average age of a	Between Groups	1771.05	3	590.351	4.052	0.007
	person who died with	Within Groups	174845.95	1200	145.705		
	Covid-19	Total	176617.01	1203			
7	% of C19 deaths	Between Groups	1593.54	3	531.180	5.160	0.002
	who were children	Within Groups	126526.11	1229	102.950		
		Total	128119.65	1232			
8	% of C19 deaths	Between Groups	18363.73	3	6121.245	8.891	0.000
	who were healthy	Within Groups	846132.00	1229	688.472		
	people between 18 -	Total	864495.74	1232			
9	% of people who	Between Groups	11531.20	3	3843.732	5.890	0.001
	recover without	Within Groups	801344.60	1228	652.561		
	medical intervention	Total	812875.79	1231			
10	% that a healthy	Between Groups	14054.00	3	4684.666	14.467	0.000
	person < 65 ends up	Within Groups	397981.67	1229	323.826		
	in ICU	Total	412035.67	1232			
11	% that a healthy	Between Groups	4845.80	3	1615.265	6.537	0.000
	person < 65 dies	Within Groups	303695.51	1229	247.108		
		Total	308541.31	1232			
12	% that a healthy	Between Groups	1661.54	3	553.845	1.165	0.322
	person < 65 never	Within Groups	584097.91	1229	475.263		
	fully recovers from	Total	585759.45	1232			
