# Supplementary material for: The new normal: Covid-19 risk perceptions and support for continuing restrictions past vaccinations
Source: PLoS One. 2022 Apr 8;17(4):e0266602. doi: 10.1371/journal.pone.0266602 (PMC8993013; doi:10.1371/journal.pone.0266602)
Supplement: S4 Table — (PDF) [file pone.0266602.s005.pdf]

Supporting information

S4 Table. One Sample *T* Test Results Per Sample.

Average age of a person who died of/with C19 (Test Value 78)

									95% CI	
	N	*M*	*SD*	*SE*	*t*	*df*	*p*	*M diff.*	*Lower*	*Upper*
A	275	63.97	11.84	0.71	-19.65	274	.0000	-14.03	-15.43	-12.62
B	294	66.97	11.76	0.69	-16.08	293	.0000	-11.03	-12.38	-9.68
C	251	64.32	11.91	0.75	-18.20	250	.0000	-13.68	-15.16	-12.20
D	384	66.11	12.56	0.64	-18.54	383	.0000	-11.89	-13.15	-10.62
E	260	60.42	12.61	0.78	-22.47	259	.0000	-17.58	-19.12	-16.04

% of C19 who were children (Test value = 1)

									95% CI	
	N	*M*	*SD*	*SE*	*t*	*df*	*p*	*M diff.*	*Lower*	*Upper*
A	275	6.69	7.60	0.46	12.41	274	.0000	5.69	4.79	6.59
B	294	8.73	9.66	0.56	13.72	293	.0000	7.73	6.62	8.84
C	254	9.96	11.80	0.74	12.10	253	.0000	8.96	7.50	10.42
D	410	9.13	10.85	0.54	15.18	409	.0000	8.13	7.08	9.19
E	263	11.64	14.03	0.87	12.30	262	.0000	10.64	8.94	12.34

% of C19 deaths for healthy people between 18 - 65 (Test value = 1)

	N	*M*	*SD*	*SE*	*t*	*df*	*p*	*M diff.*	*Lower*	*Upper*
A	275	30.88	25.51	1.54	19.42	274	.0000	29.88	26.85	32.91
B	294	35.35	27.70	1.62	21.26	293	.0000	34.35	31.17	37.53
C	254	40.19	28.51	1.79	21.91	253	.0000	39.19	35.67	42.72
D	410	30.33	24.08	1.19	24.66	409	.0000	29.33	26.99	31.66
E	263	36.36	26.12	1.61	21.96	262	.0000	35.36	32.19	38.53

% of recovery without medical intervention (Test value = 90)

	N	*M*	*SD*	*SE*	*t*	*df*	*p*	*M diff.*	*Lower*	*Upper*
A	274	67.22	26.69	1.61	-14.13	273	.0000	-22.78	-25.96	-19.61
B	294	60.01	28.06	1.64	-18.33	293	.0000	-29.99	-33.22	-26.77
C	254	62.66	22.67	1.42	-19.22	253	.0000	-27.34	-30.14	-24.54
D	410	67.07	24.53	1.21	-18.92	409	.0000	-22.93	-25.31	-20.55
E	263	67.54	25.31	1.56	-14.39	262	.0000	-22.46	-25.54	-19.39

% chance that a healthy person < 65 ends up in ICU (Test value = 1)

	N	*M*	*SD*	*SE*	*t*	*df*	*p*	*M diff.*	*Lower*	*Upper*
A	275	19.14	18.93	1.14	15.89	274	.0000	18.14	15.89	20.39
B	294	24.35	19.99	1.17	20.03	293	.0000	23.35	21.05	25.64
C	254	17.08	16.58	1.04	15.46	253	.0000	16.08	14.03	18.13

D	410	15.59	16.64	0.82	17.76	409	.0000	14.59	12.97	16.21
E	263	26.11	26.24	1.62	15.52	262	.0000	25.11	21.92	28.30

% chance that a health person < 65 dies (Test value = 1)

	N	*M*	*SD*	*SE*	*t*	*df*	*p*	*M diff.*	*Lower*	*Upper*
A	275	10.46	15.23	0.92	10.30	274	.0000	9.46	7.65	11.27
B	294	14.15	18.64	1.09	12.10	293	.0000	13.15	11.01	15.29
C	254	9.11	13.88	0.87	9.31	253	.0000	8.11	6.39	9.82
D	410	9.40	14.80	0.73	11.49	409	.0000	8.40	6.96	9.84
E	263	16.544	22.66	1.40	11.12	262	.0000	15.54	12.79	18.30
