# Supplementary material for: The new normal: Covid-19 risk perceptions and support for continuing restrictions past vaccinations
Source: PLoS One. 2022 Apr 8;17(4):e0266602. doi: 10.1371/journal.pone.0266602 (PMC8993013; doi:10.1371/journal.pone.0266602)
Supplement: S5 Table — (PDF) [file pone.0266602.s006.pdf]

Supporting information

S5 Table. Correlations and Descriptives Per Sample.

The following tables show how the core relationship between risk estimation and NNP manifests in each sample. Kindly see *Table 1* in the main manuscript for explanations behind differences in *N* (random assignment). Sample E is presented in the main manuscript because it is the only data collected in 2022.

Tables S5 (A – D). Correlations and descriptives per sample.

Sample A

				Core Estimation Indicators							*Compliance*			*Gender*	*Age*	*Ideol.*	*Consp.*	*Stats. Lit.*	
Variables	***Mean***	***SD***	***N***	*3*	*4*	*5*	*6*	*7*	*8*	*9*	*10*	*11*	*12*	*13*	*14*	*15*	*16*	*17*	*18*
1 NNP Support	4.95	1.59	140	-.37 **	.28 **	.38 **	-.42 **	.38 **	.32 **	.40 **	.14	.61 **	.54 **	-.08	-.11	-.57 **	-.32 **	.08	.05
2 RN - Fear	3.37	1.68	134	-.05	.25 **	.17	-.21 *	.27 **	.19 *	.28 **	.20 *	.32 **	.19 *	-.23 **	.24 **	-.30 **	-.04	-.22 *	.13
3 Average age of C19 death	63.97	11.84	275		-.28 **	-.32 **	.31 **	-.31 **	-.25 **	-.29 **	-.11	-.21 **	-.07	-.05	.11	.17 **	.04	.00	-.06
4 % of C19 deaths who were	6.69	7.60	275			.36 **	-.34 **	.48 **	.49 **	.39 **	.13 *	.15 *	.01	-.17 **	-.03	-.09	.13 *	-.22 **	.16 **
5 % of C19 deaths (healthy; 1	30.88	25.51	275				-.33 **	.39 **	.35 **	.39 **	.07	.28 **	.13 *	-.09	-.09	-.16 *	-.09	-.02	.07
6 % recover without medical i	67.22	26.69	274					-.46 **	-.42 **	-.41 **	-.17 **	-.27 **	-.16 **	.10	-.08	.17 **	.08	.15 *	-.15 *
7 % that a healthy person < 6	19.14	18.93	275						.86 **	.66 **	.18 **	.20 **	.05	-.15 *	-.06	-.13 *	.02	-.19 **	.14 *
8 % that a healthy person < 6	10.46	15.23	275							.70 **	.18 **	.17 **	.05	-.19 **	-.02	-.08	.07	-.19 **	.13 *
9 %healthy < 65 never fully re	17.89	21.14	275								.15 *	.21 **	.11	-.24 **	.02	-.18 **	.05	-.15 *	.07
10 Contact-tracing	1.64	1.43	272									-.01	.13 *	-.08	.06	-.06	.11	-.09	.07
11 General compliance	6.38	1.14	271										.46 **	-.08	-.11	-.40 **	-.44 **	.12	.02
12 Vaccine intent	4.22	1.30	272											.04	.07	-.37 **	-.28 **	.23 **	-.07
13 Gender (1 = male)	0.49	0.50	268												-.23 **	-.02	.06	.26 **	-.18 **
14 Age	41.24	13.28	272													.16 **	-.02	-.14 *	.11
15 Political ideology (Conserva	4.35	2.41	270														.30 **	-.14 *	.06
16 Conspiracy beliefs	2.77	2.11	275															-.08	-.04
17 Statistical literacy	1.72	1.18	275																-.68 **
18 Stat. Item 3	0.36	0.48	272																
19 Concern	.	.	0																

Sample B

				Core Estimation Indicators							*Compliance*			*Gender*	*Age*	*Ideol.*	*Consp.*	*Stats. Lit.*	
Variables	***Mean***	***SD***	***N***	*3*	*4*	*5*	*6*	*7*	*8*	*9*	*10*	*11*	*12*	*13*	*14*	*15*	*16*	*17*	*18*
1 NNP Support	5.25	1.19	148	-.23 **	.18 *	.28 **	-.27 **	.24 **	.21 *	.28 **	.19 *	.31 **	.57 **	-.06	-.10	-.24 **	-.36 **	-.12	.08
2 RN - Fear	3.57	1.65	146	-.06	.18 *	.12	-.19 *	.25 **	.31 **	.35 **	.15	.29 **	.30 **	-.17 *	.14	-.19 *	-.14	-.01	-.07
3 Average age of C19 death	66.97	11.76	294		-.27 **	-.35 **	.25 **	-.23 **	-.18 **	-.19 **	.05	.01	-.07	.05	.07	-.01	-.01	.08	-.03
4 % of C19 deaths who were	8.73	9.66	294			.43 **	-.31 **	.46 **	.45 **	.33 **	.01	.03	-.01	-.11	-.17 **	-.04	.04	-.12 *	.07
5 % of C19 deaths (healthy; 1	35.35	27.70	294				-.36 **	.46 **	.41 **	.32 **	.03	.10	.10	-.07	-.09	.02	-.08	-.18 **	.18 **
6 % recover without medical i	60.01	28.06	294					-.47 **	-.39 **	-.23 **	.01	-.05	-.12 *	.03	.12 *	-.02	-.02	.15 **	-.10
7 % that a healthy person < 6	24.35	19.99	294						.81 **	.59 **	.05	.07	.06	-.19 **	-.10	.01	.05	-.30 **	.22 **
8 % that a healthy person < 6	14.15	18.64	294							.69 **	.07	.12 *	.02	-.20 **	.00	.10	.07	-.27 **	.21 **
9 %healthy < 65 never fully re	20.23	21.91	294								.10	.21 **	.14 *	-.16 **	.00	-.03	-.01	-.17 **	.15 **
10 Contact-tracing	2.34	1.93	293									.13 *	.24 **	-.02	-.05	-.17 **	-.16 **	-.08	.03
11 General compliance	6.12	1.46	294										.32 **	-.13 *	.09	-.08	-.17 **	-.04	.00
12 Vaccine intent	4.28	1.16	294											-.02	.07	-.26 **	-.28 **	-.05	.07
13 Gender (1 = male)	0.66	0.48	291												-.10	.17 **	.00	.15 *	-.09
14 Age	29.15	10.48	293													-.06	.02	-.03	-.04
15 Political ideology (Conserva	4.24	2.09	274														.17 **	.02	.03
16 Conspiracy beliefs	2.45	1.56	293															-.06	.01
17 Statistical literacy	1.87	1.12	294																-.77 **
18 Stat. Item 3	0.38	0.49	293																
19 Concern	.	.	0																

Sample C

				Core Estimation Indicators							*Compliance*			*Gender*	*Age*	*Ideol.*	*Consp.*	*Stats. Lit.*		*Concern*
Variables	***Mean***	***SD***	***N***	*3*	*4*	*5*	*6*	*7*	*8*	*9*	*10*	*11*	*12*	*13*	*14*	*15*	*16*	*17*	*18*	*19*
1 NNP Support	5.17	1.25	253	-.12	.15 *	.22 **	-.27 **	.21 **	.14 *	.18 **	.24 **	.46 **	.53 **	.00	.01	-.16 *	-.14 *	-.13 *	.07	.60 **
2 RN - Fear																				
3 Average age of C19 death	64.32	11.91	251		-.24 **	-.41 **	.28 **	-.28 **	-.20 **	-.19 **	-.04	-.06	.00	.06	.11	-.07	-.02	.06	-.12	-.07
4 % of C19 deaths who were children	9.96	11.80	254			.32 **	-.25 **	.47 **	.50 **	.34 **	.12	-.04	-.04	-.12	-.13 *	.02	.16 *	-.15 *	.15 *	.07
5 % of C19 deaths (healthy; 18 - 65)	40.19	28.51	254				-.21 **	.34 **	.32 **	.25 **	.04	.16 *	-.04	-.06	-.15 *	.05	.00	-.08	.15 *	.28 **
6 % recover without medical intervention	62.66	22.67	254					-.40 **	-.23 **	-.23 **	-.03	-.13 *	-.09	.08	.12	.03	.06	.14 *	-.10	-.18 **
7 % that a healthy person < 65 ends up in ICU	17.08	16.58	254						.70 **	.48 **	.15 *	.03	.06	-.16 *	-.08	.04	.13 *	-.13 *	.12	.23 **
8 % that a healthy person < 65 dies	9.11	13.88	254							.53 **	.18 **	-.03	-.05	-.16 *	-.07	.01	.11	-.10	.06	.16 *
9 %healthy < 65 never fully recovers	19.48	21.56	254								.11	-.04	.11	-.15 *	-.02	-.06	-.05	-.02	.06	.24 **
10 Contact-tracing	2.60	1.99	253									.09	.16 *	-.03	.01	-.03	-.07	-.07	-.01	.23 **
11 General compliance	6.28	1.21	250										.34 **	-.04	.07	-.18 **	-.01	-.06	.02	.37 **
12 Vaccine intent	4.32	1.11	253											-.02	-.05	-.34 **	-.25 **	.04	-.02	.42 **
13 Gender (1 = male)	0.69	0.46	247												-.11	.03	-.11	.22 **	-.12	-.06
14 Age	28.56	10.37	253													.06	.10	.03	-.09	.01
15 Political ideology (Conservatism)	3.96	1.76	244														.26 **	-.04	.10	-.20 **
16 Conspiracy beliefs	2.88	1.52	253															-.12	.09	-.12
17 Statistical literacy	1.92	1.12	254																-.72 **	-.03
18 Stat. Item 3	0.31	0.47	251																	.05
19 Concern	67.98	28.24	254																	

Sample D

				Core Estimation Indicators							*Compliance*			*Gender*	*Age*	*Ideol.*	*Consp.*	*Stats. Lit.*		*Concern*
Variables	***Mean***	***SD***	***N***	*3*	*4*	*5*	*6*	*7*	*8*	*9*	*10*	*11*	*12*	*13*	*14*	*15*	*16*	*17*	*18*	*19*
1 NNP Support	4.77	1.77	406	-.34 **	.36 **	.40 **	-.41 **	.40 **	.28 **	.45 **	.74 **	.75 **	.81 **	-.26 **	.13 **	-.44 **	-.53 **	-.06	.00	.60 **
2 RN - Fear	.	.																		
3 Average age of C19 death	66.11	12.56	384		-.29 **	-.47 **	.30 **	-.34 **	-.28 **	-.33 **	-.31 **	-.30 **	-.29 **	.10	.04	.08	.13 **	.09	.02	-.23 **
4 % of C19 deaths who were children	9.13	10.85	410			.49 **	-.38 **	.43 **	.39 **	.41 **	.26 **	.28 **	.31 **	-.13 *	.02	-.11 *	-.21 **	-.10 *	.04	.21 **
5 % of C19 deaths (healthy; 18 - 65)	30.33	24.08	410				-.40 **	.49 **	.45 **	.47 **	.34 **	.36 **	.34 **	-.14 **	.00	-.12 *	-.16 **	-.12 *	.08	.29 **
6 % recover without medical intervention	67.07	24.53	410					-.48 **	-.37 **	-.44 **	-.29 **	-.30 **	-.32 **	.12 *	-.12 *	.15 **	.23 **	.07	-.07	-.24 **
7 % that a healthy person < 65 ends up in ICU	15.59	16.64	410						.64 **	.60 **	.28 **	.29 **	.31 **	-.20 **	.12 *	-.12 *	-.15 **	-.19 **	.17 **	.28 **
8 % that a healthy person < 65 dies	9.40	14.80	410							.56 **	.16 **	.18 **	.20 **	-.11 *	.08	-.12 *	-.04	-.21 **	.15 **	.17 **
9 %healthy < 65 never fully recovers	20.99	22.30	410								.29 **	.33 **	.38 **	-.23 **	-.02	-.18 **	-.23 **	-.17 **	.10	.37 **
10 Contact-tracing	5.07	2.32	401									.76 **	.72 **	-.24 **	.16 **	-.38 **	-.47 **	.06	-.06	.48 **
11 General compliance	5.78	1.85	403										.76 **	-.34 **	.05	-.38 **	-.50 **	.00	-.05	.50 **
12 Vaccine intent	4.07	1.50	396											-.20 **	.04	-.38 **	-.58 **	.05	-.07	.49 **
13 Gender (1 = male)	0.28	0.45	389												.00	.20 **	.11 *	.16 **	-.10 *	-.24 **
14 Age	51.53	15.78	395													.10	-.04	-.18 **	.14 **	.07
15 Political ideology (Conservatism)	3.80	1.91	350														.34 **	.00	.00	-.25 **
16 Conspiracy beliefs	2.85	1.88	408															-.07	.11 *	-.29 **
17 Statistical literacy	1.96	1.12	410																-.70 **	-.07
18 Stat. Item 3	0.28	0.45	388																	.03
19 Concern	57.58	38.15	404																	

* $p < .01$; ** $p < .01$
