# Supplementary material for: The new normal: Covid-19 risk perceptions and support for continuing restrictions past vaccinations
Source: PLoS One. 2022 Apr 8;17(4):e0266602. doi: 10.1371/journal.pone.0266602 (PMC8993013; doi:10.1371/journal.pone.0266602)
Supplement: S6 Table — (PDF) [file pone.0266602.s007.pdf]

Supporting information

S6 Table. Regression Results per Sample.

	All Samples		Sample A		Sample B		Sample C		Sample D	
Predictors	*B*	*p*	*B*	*p*	*B*	*p*	*B*	*p*	*B*	*p*
1 Average age of C19 death	-0.01	.002	-0.02	.047	-0.01	.129	0.00	.639	-0.02	.016
2 % of C19 deaths: Children	0.01	.155	0.00	.871	0.00	.952	0.00	.746	0.02	.076
3 % of C19 deaths: Healthy between 18 - 65	0.01	.000	0.01	.026	0.01	.067	0.01	.032	0.01	.011
4 % recover without intervention	-0.01	.000	-0.01	.010	-0.01	.090	-0.01	.005	-0.01	.001
5 % that a healthy person < 65 is hospitalized	0.01	.011	0.01	.406	0.00	.889	0.01	.433	0.01	.058
6 % that a healthy person < 65 dies	-0.01	.006	-0.01	.613	-0.01	.398	-0.01	.543	-0.02	.033
7 % that a healthy person < 65 never fully recovers	0.01	.000	0.01	.169	0.01	.019	0.01	.240	0.02	.000
F (df)	42.05 (7, 911)		9.04 (7, 192)		4.03 (7, 147)		4.23 (7, 243)		25.62 (7,372)	
	p < .001		*p* < .001		*p* < .001		*p* < .001		*p* < .001	
