# Supplementary material for: The new normal: Covid-19 risk perceptions and support for continuing restrictions past vaccinations
Source: PLoS One. 2022 Apr 8;17(4):e0266602. doi: 10.1371/journal.pone.0266602 (PMC8993013; doi:10.1371/journal.pone.0266602)
Supplement: S7 Table — (PDF) [file pone.0266602.s008.pdf]

Supporting information

S7 Table (A - D): Regression Results with Controls per Sample.

Sample A

		Step 1			Step 2		
	Predictors	*B*	*SE*	*p*	*B*	*SE*	*p*
1	Gender	-0.14	0.23	.533	0.15	0.21	.484
2	Ideology (Conservatism)	-0.33	0.05	.000	-0.27	0.04	.000
3	Conspiracy beliefs	-0.07	0.05	.188	-0.08	0.05	.084
4	Age				-0.01	0.01	.113
5	% of C19 deaths: Children				-0.01	0.01	.629
6	% of C19 deaths: Healthy between 18 - 65				0.01	0.00	.024
7	% recover without intervention				-0.01	0.00	.011
8	% that a healthy person < 65 is hospitalized				0.01	0.01	.388
9	% that a healthy person < 65 dies				0.01	0.01	.480
10	% that a healthy person < 65 never fully recovers						
	F (df)	21.77 (3, 133)			14.35 (10, 126)		
		$p < .001$			$p < .001$		
	$R^2 \Delta$				0.20 ($p < .001$)		
	Model R^2	.33			.53		

Gender (1 = *male* ; 0 = *female*)

Sample B

	Predictors	Step 1 B	Step 1 SE	Step 1 p	Step 2 B	Step 2 SE	Step 2 p
1	Gender	0.00	0.20	.995	0.23	0.19	.239
2	Ideology (Conservatism)	-0.11	0.05	.023	-0.12	0.05	.015
3	Conspiracy beliefs	-0.22	0.06	.001	-0.20	0.06	.001
4	Age				-0.02	0.01	.049
5	% of C19 deaths: Children				-0.01	0.01	.654
6	% of C19 deaths: Healthy between 18 - 65				0.01	0.00	.170
7	% recover without intervention				-0.01	0.00	.061
8	% that a healthy person < 65 is hospitalized				0.00	0.01	.933
9	% that a healthy person < 65 dies				0.00	0.01	.896
10	% that a healthy person < 65 never fully recovers				0.01	0.01	.052
	F (df)	7.21 (3, 135)			5.56 (10, 128)		
			$p < .001$			$p < .001$	
	$R^2 \Delta$				0.17 ($p < .001$)		
	Model R^2		.14			.30	

Gender (1 = *male* ; 0 = *female*)

Sample C

	Predictors	Step 1 B	Step 1 SE	Step 1 p	Step 2 B	Step 2 SE	Step 2 p
1	Gender	0.02	0.18	.910	0.23	0.19	.239
2	Ideology (Conservatism)	-0.12	0.05	.016	-0.12	0.05	.015
3	Conspiracy beliefs	-0.07	0.06	.217	-0.20	0.06	.001
4	Age				-0.02	0.01	.049
5	% of C19 deaths: Children				-0.01	0.01	.654
6	% of C19 deaths: Healthy between 18 - 65				0.01	0.00	.170
7	% recover without intervention				-0.01	0.00	.061
8	% that a healthy person < 65 is hospitalized				0.00	0.01	.933
9	% that a healthy person < 65 dies				0.00	0.01	.896
10	% that a healthy person < 65 never fully recovers				0.01	0.01	.052
	F (df)	3.34 (3, 232)			4.36 (10, 225)		
			$p = .019$			$p < .001$	
	$R^2 \Delta$				0.12 ($p < .001$)		
	Model R^2		.04			.16	

Gender (1 = *male* ; 0 = *female*)

Sample D

Predictors	Step 1 *B*	Step 1 *SE*	Step 1 *p*	Step 2 *B*	Step 2 *SE*	Step 2 *p*
1 Gender	-0.63	0.17	.000	-0.31	0.16	.054
2 Ideology (Conservatism)	-0.25	0.04	.000	-0.24	0.04	.000
3 Conspiracy beliefs	-0.40	0.05	.000	-0.31	0.04	.000
4 Age				-0.01	0.01	.079
5 % of C19 deaths: Children				0.01	0.01	.179
6 % of C19 deaths: Healthy between 18 - 65				0.01	0.00	.041
7 % recover without intervention				-0.01	0.00	.020
8 % that a healthy person < 65 is hospitalized				0.01	0.01	.060
9 % that a healthy person < 65 dies				-0.01	0.01	.293
10 % that a healthy person < 65 never fully recovers				0.01	0.00	.030
F (df)	64.95 (3, 310) $p < .001$			33.75 (10, 303) $p < .001$		
$R^2 \Delta$				0.14 ($p < .001$)		
Model R^2	.39			.53		

Gender (1 = *male*; 0 = *female*)
