# Supplementary material for: The new normal: Covid-19 risk perceptions and support for continuing restrictions past vaccinations
Source: PLoS One. 2022 Apr 8;17(4):e0266602. doi: 10.1371/journal.pone.0266602 (PMC8993013; doi:10.1371/journal.pone.0266602)
Supplement: S8 Table — (PDF) [file pone.0266602.s009.pdf]

Supporting information

RN-Fear is a 3-item, affect-based dependent variable to capture participants' fear of returning to 'normal' once all the vulnerable groups have been vaccinated and once everybody has had a chance to receive their vaccines. However, this variable was only administered in initial waves (Samples A and B). Half of the participants received RN-Fear and the other half received the core DV (NNP support). Table 4 SOM shows regression results using Samples A and B only.

S8 Table. Predicting RN-fear with Controls.

		Step 1			Step 2		
Predictors		*B*	*SE*	*p*	*B*	*SE*	*p*
1	Gender	-.67	.20	.000	-.55	.19	.005
2	Ideology	-.17	.05	.000	-.14	.04	.002
3	Conspirac	-.10	.06	.099	-.12	.06	.037
4	Average age of Covid				.01	.01	.264
5	% of C19 deaths: Children				.02	.02	.331
6	% of C19 deaths: Healthy between 18 - 65				.00	.00	.413
7	% recover without intervention				.00	.00	.419
8	% that a healthy person < 65 ends up in ICU				.01	.01	.461
9	% that a healthy person < 65 dies				.00	.01	.867
10	% that a healthy person < 65 never fully recovers				.02	.01	.001
	F (df)		9.97 (3, 255) $p < .001$			6.66 (10, 248) $p < .001$	
	R^2 Δ					.11	
	Model R^2		.11			.21	

Gender (1 = *male* ; 0 = *female*)
