# Supplementary material for: The new normal: Covid-19 risk perceptions and support for continuing restrictions past vaccinations
Source: PLoS One. 2022 Apr 8;17(4):e0266602. doi: 10.1371/journal.pone.0266602 (PMC8993013; doi:10.1371/journal.pone.0266602)
Supplement: S9 Table — (PDF) [file pone.0266602.s010.pdf]

Supporting information

S9 Table. Descriptive statistics and correlations without outliers (+/2 SD; Samples A - D). The following table shows correlations between Covid-19 estimation and new normal support (NNP and RN: Fear) after removing the outliers. Outliers were identified based on 2 standard deviations. Specifically, all participants' responses to core indicators were standardized within each sample (i.e., Z scores were generated relative to other participants in each sample). Responses from participants who over-estimated one or more core indicators beyond 2 SDs were eliminated. The results, however, remain largely the same.

				Contact	*Compliance*	*Vaccine*			*Core C19 Estimation Indicators*					*Conspir.*	*Stats.*	*Gender*	*Age*	*Ideology*	*Concern*
Variables	*Mean*	*SD*	N	3	4	5	6	7	8	9	10	11	12	13	14	15	16	17	18
Core DVs: Restrictions Past Vaccinations																			
1 NNP support	4.83	1.60	744	0.37 **	0.65 **	0.71 **	-0.32 **	0.31 **	0.34 **	-0.36 **	0.31 **	0.26 **	0.34 **	-0.42 **	-0.04	-0.05	-0.06	-0.38 **	0.63 **
2 RN-fear	3.30	1.66	217	0.21 **	0.26 **	0.28 **	-0.02	0.17 *	0.11	-0.06	0.21 **	0.18 **	0.30 **	-0.14 *	-0.04	-0.20 **	0.14 *	-0.27 **	
3 Contact-tracing	3.10	2.43	956		0.27 **	0.32 **	-0.09 **	0.20 **	0.07 *	-0.09 **	0.04	0.05	0.20 **	-0.21 **	0.04	-0.23 **	0.31 **	-0.22 **	0.28 **
4 General compliance	6.02	1.59	954			0.59 **	-0.20 **	0.20 **	0.22 **	-0.21 **	0.16 **	0.13 **	0.21 **	-0.37 **	-0.01	-0.12 **	-0.08 *	-0.29 **	0.51 **
5 Vaccine intent	4.18	1.34	954				-0.19 **	0.19 **	0.20 **	-0.22 **	0.18 **	0.14 **	0.27 **	-0.43 **	0.07 *	-0.03	-0.05	-0.36 **	0.49 **
6 Average age of C19 death	67.52	9.91	943					-0.34 **	-0.42 **	0.31 **	-0.27 **	-0.25 **	-0.22 **	0.06	0.01	-0.03	0.07 *	0.07 *	-0.20 **
7 % of C19 deaths who were children	6.36	6.16	965						0.52 **	-0.31 **	0.37 **	0.36 **	0.30 **	-0.06	-0.08 *	-0.13 **	-0.03	-0.10 **	0.24 **
8 % of C19 deaths - healthy (18 - 65)	27.28	22.39	965							-0.36 **	0.40 **	0.38 **	0.30 **	-0.09 **	-0.09 **	-0.03	-0.17 **	-0.07 *	0.30 **
9 % recover without medical intervention	70.25	21.51	964								-0.41 **	-0.33 **	-0.31 **	0.12 **	0.12 **	0.02	0.08 *	0.08 *	-0.28 **
10 % that a healthy person < 65 ends up in ICU	14.15	12.99	965									0.73 **	0.44 **	-0.06	-0.16 **	-0.08 *	-0.13 **	-0.08 *	0.26 **
11 % that a healthy person < 65 dies	6.28	7.92	965										0.48 **	-0.03	-0.16 **	-0.12 **	-0.06 *	-0.01	0.17 **
12 % healthy < 65 never recovers from long C19	14.29	14.87	965											-0.15 **	-0.06	-0.18 **	-0.02	-0.15 **	0.32 **
13 Conspiracy beliefs	2.75	1.81	962												-0.06	0.03	0.03	0.28 **	-0.28 **
14 Statistics literacy	1.95	1.12	965													0.16 **	-0.07 *	-0.04	-0.02
15 Gender	0.52	0.50	943														-0.28 **	0.09 **	-0.06
16 Age	39.18	16.11	951															0.03	-0.06
17 Political ideology	4.10	2.04	893																-0.22 **
18 Concern over contracting C19	59.68	35.17	526																

* $p < .01$; ** $p < .01$; Gender (1 = *male*; 0 = *female*)
