# Supplementary material for: The new normal: Covid-19 risk perceptions and support for continuing restrictions past vaccinations
Source: PLoS One. 2022 Apr 8;17(4):e0266602. doi: 10.1371/journal.pone.0266602 (PMC8993013; doi:10.1371/journal.pone.0266602)
Supplement: S1 File — (PDF) [file pone.0266602.s011.pdf]

Supporting information

Additional Results File

Alternative Risk Indicator 1: Perceptions of Global Death Toll

In contrast to the above-mentioned indicators of health risks, here the participants reflected on millions of Covid-19 deaths around the world and they were asked to indicate how many of those individuals would still be alive, if it were not for Covid. This question aludes to the difficulty to quantify deaths or hospitalizations that occur with Covid-19 or of Covid-19. Because there is no definitive answer to this question, I treat this variable as an exploratory indicator of participants' beliefs, and do not include it in *t* test analysis of mid-point difference. Specifically, participants were asked to:

> "Consider the millions of individuals who have died with Covid-19 across the world. What percentage of those individuals who died would STILL BE ALIVE, if it weren't for Covid-19? Select lower % numbers if you believe that almost nobody would still be alive (i.e., they would die of other causes or old age around the same time). Select higher % numbers if you believe that almost everybody would still be alive."

Participants provided their estimate on a slider scale without an anchored pointer. Labels were positioned above the scale noting 0 (*almost nobody*) to 100 (*almost everybody*). Belief in the percentage of people who would still be alive (M = 67.90, DS = 24.30) was strongly and positively related to NNP support (r = .50, p < .001), but not RN-fear (r = .08, p = .31).

Alternative Indicators: 1,000 Base

3.1. Alternative Risk Indicator 1: Estimates based on 1,000 & NNP Support

Participants made outcome estimation based on 1,000 people (instead of 100 corresponding to 100%). 65 years of age was used as the cut-off which is consistent with CDC cut-off for Covid-19 risk communication {CDC, 2021 #84;Ioannidis, 2021 #49;Ioannidis, 2020 #130237}. Drawing conclusions by using 1,000 (vs. only 100) offers greater ability to detect under- or over-estimates in interpretation. The Qualtrics mode was set up in such a way that participants were forced to distribute risks so that they add up the numbers to 1,000. While this approach has limitations in that a Covid-19 patient can suffer from long Covid-19 and be hospitalized, I chose it as a group-level complement to individual-based questions above. Instructions were:

> "Consider 1,000 healthy, fit, and non-elderly (under 65 years of age) individuals who have no major health conditions. If they contract Covid-19, indicate how many of them will experience outcomes noted below: 1) die, 2) end up in intensive care unit (ICU), 3) be hospitalized (non-ICU), 4) suffer debilitating long Covid and not be able to work, and 5) fully recover within 2 months."

Participants adjusted the sliding bars to show how many of those 1,000 people will experience each of those outcomes. The distribution had to amount to 1,000. Participants provided their response on a slider scale ranging from 0 (*almost nobody*) to 1000 (*almost everybody*). The scale had no anchor points which could have clouded participants' judgment.

In line with the core items, over-estimation of negative outcomes (being hospitalized), and under-estimation of positive outcomes (recovering) led to stronger support of NNPs.

Table S10. 1000-based estimate results.

Estimation of Outcomes	*M (for 1,000)*	*% Equivalent*	*SD*	*N*	*NNP*	*Fear*	*Tracing*	*Comp.*	*Vaccine*
					Correlation with				
1 Die	**48.49**	***4.85%***	63.65	278	.14	.16	.12 *	.06	.05
2 End up in ICU	**92.87**	***9.29%***	78.79	278	.23 **	.14	.07	.04	.10
3 Be hospitalized	**134.64**	***13.46%***	95.07	278	.24 **	.08	.07	.04	.03
4 Suffer from long Covid	**114.01**	***11.40%***	96.33	278	.13	.02	.03	.00	.02
5 Fully recover within 2 months	**609.99**	***61.00%***	245.53	278	-.25 ***	-.13	-.09	-.04	-.06

* = $p < .05$, ** = $p < .01$, *** = $p < .001$

Alternative Indicator: Perceived Scientific Consensus

Preliminary Administration: Samples A and B

In Samples A and B, a sub-set of PSC items was administered to participants at random. The objective was to obtain a preliminary understanding of the relationship between perceived scientific consensus regarding Covid-19 risks and participants' support for NNP. Therefore, the following results from Samples A and B should be interpreted with caution due to uneven sample size.

Table S11. Item-based relationship between PSC and NNP/RN-Fear (Samples A and B)

PSC Items		*r with NNP*		*N*	*r with RN-Fear*		*N*
1	Getting a COVID-19 vaccine helps keep you from getting seriously ill even if you do get COVID-19.	**.67**	**	*60*	***.31***		*39*
2	Benefits of lock-downs outweigh the costs of failing to contain Covid-19.	**.64**	**	*149*	***.44***	**	*129*
3	Masks are effective in protecting people against Covid-19.	**.77**	**	*148*	***.26***	**	*129*
4	In case of community outbreaks, people should wear masks ANY time they are outside, even if they are by themselves (e.g., driving or hiking).	**.55**	**	*150*	***.13***		*130*
5	When a person is reported as Covid-19 death, it is clear that Covid-19 was the main cause of death.	**.59**	**	*150*	***.32***	**	*130*
6	New variants spread faster AND are also far deadlier than the original variant.	**.68**	**	*51*	***.46***	**	*47*
7	In case of community outbreaks, outdoor spaces (beaches or parks) should be closed.	**.54**	**	*147*	***.31***	**	*129*
8	Elimination (Zero-Covid) is the best strategy.	**.34**	**	*57*	***.32***	*	*60*
9	People without Covid-19 symptoms should wear masks to minimize the spread of Covid-19.	**.66**	**	*52*	***.23***		*61*
10	People should disinfect their groceries to reduce their chances of contracting Covid-19.	**.42**		*150*	***.09***		*130*
11	Hydroxychloroquine is effective at preventing illness and death from Covid-19.	**-.58**	**	*48*	***-.31***		*41*
12	Covid-19 can be transmitted through mosquitoes.	**-.19**		*66*	***.31***	*	*46*
13	Benefits of children wearing masks in schools outweigh the risks of Covid-19.	**.77**	**	*57*	***.26***		*51*
14	People who test positive for Covid-19 but have no symptoms still account for a significant portion of Covid-19 infections.	**.33**	*	*59*	***.35***	*	*40*

Sources for additional items:

- Hydroxychloroquine is effective at preventing illness and death from Covid-19. ***False.*** {Axfors, 2021 #131087}
- Almost everybody has a health condition that puts them at high risk of getting seriously ill from Covid-19. ***False.*****{CDC, 2021 #131066;de Siqueira, 2020 #130775;Jin, 2021 #130918}**
- Covid-19 can be transmitted through mosquitoes. ***False. {WHO, 2020 #130862}***
- Benefits of children wearing masks in schools outweigh the risks of Covid-19. ***NA***
- People who test positive for Covid-19 but have no symptoms still account for a significant portion of Covid-19 infections. ***NA***

Exploratory Analysis: Comparing Participants Who Deny Covid-19 vs. Those Who Do Not

The following table shows results from comparing participants who have *strongly disagreed* with the statement "Covid-19 is not real; it doesn't exist" (majority of all participants; labeled as NA) and participants who have selected '*disagree*' or higher (labeled as Consp). The starkest differences among the two groups emerge in their current self-report compliance. Participants who do not think that Covid-19 is real scored less on health-minded behaviours and they tend to be more conservative.

Table S12. Covid-19 denialism.

Variables		*N*	*M*	*SD*	*Equality of Variances* *F*	*p*	*t*	*df*	*p*	95% CI
What is the average age of a person who died with Covid-19?	Consp.	100	**67.99**	15.35	13.74	.00	2.15	919	.031	[.25, 5.34]
	NA	821	**65.19**	11.82			1.76	114	.081	
% of C19 deaths who were children	Consp.	106	**7.93**	11.54	1.16	.28	-.84	947	.400	[-3.07, 1.23]
	NA	843	**8.85**	10.49			-.78	128	.436	
% of C19 deaths who were healthy people between 18 - 65	Consp.	106	**22.96**	25.44	.11	.74	-4.44	947	.000	[-17.26, -6.69]
	NA	843	**34.94**	26.24			-4.55	135	.000	
% of people who recover without medical intervention	Consp.	106	**72.06**	26.28	1.23	.27	3.07	947	.002	[2.86, 12.96]
	NA	843	**64.15**	24.80			2.94	130	.004	
% that a healthy person < 65 ends up in ICU	Consp.	106	**15.45**	20.23	4.01	.05	-1.30	947	.194	[-5.98, 1.21]
	NA	843	**17.84**	17.46			-1.16	125	.249	
% that a healthy person < 65 dies	Consp.	106	**10.71**	20.35	9.68	.00	.59	947	.553	[-2.17, 4.05]
	NA	843	**9.77**	14.62			.46	119	.646	
% that a healthy person < 65 never fully recovers from Long Covid	Consp.	106	**15.52**	22.02	.08	.77	-2.09	947	.037	[-9.09, -.29]
	NA	843	**20.21**	21.70			-2.07	132	.040	
NNP	Consp.	96	**3.00**	1.84	47.20	.00	-14.52	792	.000	[-2.51, -1.91]
	NA	698	**5.21**	1.33			-11.37	109	.000	
Contact-tracing	Consp.	106	**2.27**	1.92	73.52	.00	-5.38	936	.000	[-1.87, -.87]
	NA	832	**3.64**	2.53			-6.65	156	.000	
Compliance with Covid-19 mandates	Consp.	106	**4.10**	2.26	171.19	.00	-15.56	934	.000	[-2.49, -1.93]
	NA	830	**6.32**	1.22			-9.90	113	.000	
Vaccine intent	Consp.	105	**2.55**	1.67	81.52	.00	-14.47	930	.000	[-2.07, -1.57]
	NA	827	**4.37**	1.15			-10.86	117	.000	
Ideology	Consp.	88	**5.24**	1.93	1.93	.17	6.10	862	.000	[.92, 1.78]
	NA	776	**3.89**	1.97			6.20	109	.000	

Exploratory Variables

Moral Emotions / New Zealand / New Zealand Envy

Sample D (ANZ) participants were asked to indicate the extent to which thinking about how NZ has handled Covid-19 makes participants feel: 1) warmth, 2) awe, 3) compassion, 4) goodness, and 5) kindness. 1 = *strongly disagree*; 7 = *strongly agree* (α = .95). Those moral emotions {Haidt, 2003 #3873} were explored to examine whether individual-level overestimation of covid-19 risk influences participants' moral admiration with New Zealand, which has received significant international praise for their early pandemic response. In Sample C, people were asked to think about a country like NZ and how they have handled Covid-19 and answer the same questions. The drawback of administering this question to Sample C (non-NZ) was that the question assumed participants knew about NZ. The underlying relationships between estimation and New Zealand Elevation/Envy were the same.

Table S13. Predicting moral elevation in New Zealand.

Variables	Sample D		Sample D	
	r	*p*	*r*	*p*
1 NNP support	.78	***	.45	***
3 Contact tracing	.65	***	.22	***
4 Compliance	.66	***	.33	***
5 Vaccine intent	.68	***	.28	***
6 What is the average age of a person who died with Covid-19?	-.26	***	-.01	
7 % of C19 deaths who were children	.25	***	.07	
8 % of C19 deaths who were healthy people between 18 - 65	.35	***	.09	
9 % of people who recover without medical intervention	-.30	***	.07	
10 % that a healthy person < 65 ends up in ICU	.27	***	.02	
11 % that a healthy person < 65 dies	.19	***	.03	
12 % that a healthy person < 65 never fully recovers from Long Covid	.31	***	.13	*

Covid-19 Risk Information Censorship

Consider the people deciding whether responses to Covid-19 are worth the cost. Indicate the extent to which you believe that each of these groups should help governments decide what to do:

0 = *should NOT help decide*; 6 = *should decide*

1. Health scientists and practitioners
2. Non-health scientists (e.g., economists, social scientists)
3. Business owners, general public

REFERENCES

1. CDC. Covid-19 mortality overview: Center for Disease Control and Prevention; 2021 [updated April 23, 2021]. Available from: https://www.cdc.gov/nchs/covid19/mortality-overview.htm.

2. Ioannidis JPA, Axfors C, Contopoulos-Ioannidis DG. Second versus first wave of covid-19 deaths: Shifts in age distribution and in nursing home fatalities. Environmental Research. 2021;195:110856.

3. Ioannidis JPA, Axfors C, Contopoulos-Ioannidis DG. Population-level covid-19 mortality risk for non-elderly individuals overall and for non-elderly individuals without underlying diseases in pandemic epicenters. Environ Res. 2020;188:109890-.

4. Axfors C, Schmitt AM, Janiaud P, van’t Hooft J, Abd-Elsalam S, Abdo EF, et al. Mortality outcomes with hydroxychloroquine and chloroquine in COVID-19 from an international collaborative meta-analysis of randomized trials. Nature Communications. 2021;12(1):2349.

5. CDC. Weekly updates by select demographic and geographic characteristics: Center for Disease Control and Prevention; 2021 [Continuously updated]. Available from: https://www.cdc.gov/nchs/nvss/vsrr/covid_weekly/index.htm#Comorbidities.

6. de Siqueira JVV, Almeida LG, Zica BO, Brum IB, Barceló A, de Siqueira Galil AG. Impact of obesity on hospitalizations and mortality, due to COVID-19: A systematic review. Obes Res Clin Pract. 2020;14(5):398-403.

7. Jin J, Agarwala N, Kundu P, Harvey B, Zhang Y, Wallace E, et al. Individual and community-level risk for COVID-19 mortality in the United States. Nature Medicine. 2021;27(2):264-9.

8. WHO. Immunizing the public against misinformation: World Health Organization; 2020 [updated August 25, 2020]. Available from: https://www.who.int/news-room/feature-stories/detail/immunizing-the-public-against-misinformation.

9. Haidt J. The moral emotions. In: Davidson RJ, Scherer KR, Goldsmith HH, editors. Handbook of affective sciences. Oxford: Oxford University Press; 2003. p. 852-70.
