# Supplementary material for: The new normal: Covid-19 risk perceptions and support for continuing restrictions past vaccinations
Source: PLoS One. 2022 Apr 8;17(4):e0266602. doi: 10.1371/journal.pone.0266602 (PMC8993013; doi:10.1371/journal.pone.0266602)
Supplement: Attachment — Submitted filename: PLOSONE R&R Response Letter.docx [file pone.0266602.s012.docx]

**Dr. Hanna Landenmark**

*Senior Editor PLOS ONE*

**PONE-D-21-17141**

**Covid-19 Risk Perceptions, Over-estimations Predict Support for the ‘New Normal’ and Continuing Restrictions Past Vaccinations**

Thank you so much for inviting me to resubmit my manuscript. I was delighted to be given a chance to revise and strengthen my work. I acknowledge fully that my empirical package is now far more defensible, thanks to your and the review team’s clear and converging suggestions.

**In summary, I made the following major changes:**

1. **I provided far more detail about the methodology.** I clarified my data collection strategies, and I provided better explanations of my assessments. I also noted that the study was reviewed and approved by the University of Otago Human Ethics Committee Reference#D20-088.
2. **I collected additional data from January, 2022 to ensure my results remain relevant.** While not requested by you or the reviewers, this new Sample E addresses some of the challenges I have encountered in my prior data collection and helps this manuscript remain current.
3. **I reframed the study presentation.** Per your and the review team’s suggestions, I revised the scope of my work which is now evident in the introduction and the discussion.
4. **I identified the implications of this research.** I took greater care to describe the impact of my findings. I now provide a lengthier and more nuanced *Limitations* section and I crafted a new component of the discussion (*Beyond Data*), in which I specifically outlined new areas of consideration.
5. **I copyedited the manuscript.** I followed your and reviewers’ suggestions, so I made thorough edits to ensure consistency in my writing and presentation.

Below I explain how I have addressed your and the reviewers’ suggestions. I am confident that the review team’s comments strengthened this manuscript. I hope you will agree and that your highly interdisciplinary PLOS ONE readership will see value in this work.

Thank you once again for your continuing support. I look forward to your feedback.

**REVIEWER #1 INPUT**

The article clearly defines the problem it intends to analyze. However, the review of the investigations previously conducted returns a state-of-the-art too partial. In my humble opinion, the authors do not clearly identify the contradictions and inconsistencies present in the literature on the subject, ending up by suggesting in a biased and excessively monocausal way the possible steps to solve the problem.

There is now an enormous number of studies on the most varied repercussions that the current pandemic is having on the global population, as some of the examples below testify.

Okabe-Miyamoto, K., Folk, D., Lyubomirsky, S., & Dunn, E. W. (2021). Changes in social connection during COVID-19 social distancing: It’s not (household) size that matters, it’s who you’re with. Plos one, 16(1), e0245009. https://doi.org/10.1371/journal.pone.0245009

Luchetti, M. , Lee J.H. , Aschwanden, D. , Sesker , A., Strickhouser , J., Terracciano, A. & Sutin, A. (2020) . The trajectory of loneliness in response to COVID-19. American Psychologist. Advanceonlinepublication.http://dx.doi.org/10.1037/amp0000690

Tull, M. T., Edmonds, K. A., Scamaldo, K. M., Richmond, J. R., Rose, J. P., & Gratz, K. L. (2020). Psychological outcomes associated with stay-at-home orders and the perceived impact of COVID-19 on daily life. Psychiatry Research, 289, <https://doi.org/10.1016/j.psychres.2020.113098>

Even approaches different from attitudinal, psychometric and HIP ones, such as the special issue mentioned below, testify that the impact of the pandemic is multifaceted and impossible to reduce to a mere problem of “accurate information”. While recognizing the central role that the society cannot be simplistically reduced to a source of information, but must be considered a source of meaning. On issues of their interest, people construct questions and search for answers, rather than merely perceiving and processing information derived from the social context. See for example:

Papers on Social Representations - Special issue (2020). Social Representations of Covid-19: Rethinking the Pandemic’s Reality and Social Representations [Vol 29, No 2]

The article certainly proposes an interesting vision of the impact of the current pandemic on the population, imagining an extremely original way to help people to cope with it, however, my impression is that the author has a vision of some dimensions of the problem too unilinear.

In general, it seems to me that the paper technically sounds, that the claims are convincing, appropriately discussed in the context of previous literature and fully supported by the empirical data. Overall, the paper is very well organized, clearly written and sharp in its observations regarding the consequences of the subject of study.

***Authors’ Reply*:**

Thank so much for your positive assessments of my work. I am delighted to see that you and other reviewers saw merit in this idea. I do agree with you that there are numerous potential explanations for the effects that I have observed, and I acknowledge that it is challenging to say with certainty whether risk over-estimation of Covid-19 outcomes is a predictor of new normal support, or there are other, untapped explanations.

In the end, any single manuscript can only assess a select set of questions. I now clarify throughout the manuscript that my aims are not to test a complete model of all the possible antecedents to *new normal* support. Instead, my aims are more modest as I seek to test only one relationship out of many.

I now state more clearly that this is one of my limitations:

“Third, the study only assessed risk estimation as one out of many potential predictors of continuing restrictions post-vaccinations. While I chose this predictor deliberately based on recent evidence that people over-estimate Covid-19 risks, there are other reasons that could presumably influence participants’ support for ongoing restrictions (e.g., participants’ general pro-sociality, their perceptions of greater risk to elderly, and trust in governments).”

“Fifth, I assessed participants’ concern over contracting Covid-19 in all samples. However, I inadequately explored its potential influence because it is not obvious whether its relationship is one of a predictor or a dependent variable. To be sure, that challenge is present in my other interpretations due to cross-sectional nature of this data. From a theoretical perspective, however, it is plausible that concern over contracting Covid-19 leads one to selectively consume fear-based information causing them to over-inflate the risks; or, it is equally plausible that over-inflation of risks cause one to be more concerned. Exploratory path analyses support both speculations. Because the nature of the two variables is likely mutually reinforcing, future research can acknowledge that concern over Covid-19 has value and consider how it relates to risk-estimation and NNP endorsement over time.”

I hope that by testing a single relationship defensibly, providing evidence using converging metrics, making my data available, and its imperfections known, this study will form one small pixel of the available knowledge pertaining to this pandemic. Once again, thank you so much for encouraging me to state my aims more precisely.

**REVIEWER #2 INPUT**

This manuscript – essentially exploring extent to which people’s estimation of COVID-19 risks influences their support for living with restrictions in the new normal – is one of the most enjoyable pieces of scientific writing I have reviewed since the start of the pandemic. As such, the study is built on a thoughtful rationale and a sound base in theory. The methodological approach is rigorous (but not without its limitations that have been sufficiently addressed in the discussion) to the extent to which the research questions are answered, and is explained in sufficient detail to merit replication. The discussion focuses on the key findings and while some of the points are mildly provocative (which makes this work even more engaging), the manuscript is careful in its claims without extrapolating beyond the scope of the data. Overall, I am pleased to recommend this manuscript for publication should the very minor amendments listed below be made.

***Author’s Reply*:**

Thank you so much for such a positive assessment of my work. Your encouraging words and your pointed critique motivated me to revise this manuscript thoroughly and make it a worthy addition to the pandemic literature.

1) There’s a missing word in this sentence (pg. 10, last paragraph): “The secondary measure of (?) was a 3-item affect-based variable reflecting…”

***Author’s Reply*:**

Fixed as follows (the missing words are underlined):

The secondary measure of support for the new normal was a 3-item affect-based variable reflecting participants’ fears of returning to normal under same vaccination conditions (labelled as RN-Fear and administered to Samples A and B only).

2) In Table 4, the asterisk is not qualified

***Author’s Reply*:**

Thank you for bringing it to my attention. I removed this asterisks and instead, I provided a *Notes* section under this and other tables.

3) Its not clear why the Cronbach’s alpha scores for the RN-Fear scale have not been presented.

***Author’s Reply*:**

This information is noted under “Fear of Abandoning Covid-19 Restrictions (Samples A and B only)”:

Due to their high internal consistency (Cronbach’ α coefficients were .96 and .92 for Samples A and B, respectively), items were collapsed to form a single scale labelled RN-fear of returning to normal.

4) Table 5: PSC needs to be elaborated.

***Author’s Reply*:**

Done. I expanded this description and provided it within Tables 5 and 6 titles and notes.

5) While the authors were mindful to ask participants about “Concern over Contracting COVID-19”, it is unclear why they were not asked directly about whether they had previously contracted COVID-19 or not? Disease experience/disease history could be a critical factor in shaping risk perceptions around the ‘new normal’.

***Author’s Reply*:**

That is a good point. During my 2021-based data collections (April), I avoided asking direct questions about participants’ health. Perhaps erroneously, I reasoned that asking about their Covid-19 recovery status would be inappropriate and I did not file it with my ethics application. Therefore, I wanted to assess their concern over contracting Covid-19 without intruding.

However, much has changed over the last few months. While none of the reviewers asked me to do so, I wanted to replicate my general relationship (risk over-estimation and NNP endorsement) with a newer sample. Therefore, I now provide Sample E data (Mturk-based responses collected in January 2022). Given that there is significantly more discussion about Covid-19 recovery and vaccines, I filed an amendment with my University’s Human Subjects Review Board and I asked two questions: 1) whether the participants had Covid (*yes*, *no*, *I do not wish to answer*)*,* and 2) whether they are vaccinated (*yes*, *no*, *I do not wish to answer*).

I now report this information in general correlations table (Table 9; Sample E) and regression. Data suggested that people who have had Covid-19 and who are unvaccinated are less likely to support the new normal. Overall, the effect of estimations on NNP remains even after controlling for participants’ vaccination and recovery status.

6) In pg. 16, the word ‘limitations’ is spelt without the ‘o’.

***Author’s Reply*:**

I corrected this embarrassing error. Thank you.

7) In pg. 19, it needs to be changed to “both 5-item scales” (plural)

***Author’s Reply*:**

Done. Thank you.

8) In pg. 34, the sentence reads “only if they believed there is scientific evidence on issues that are actually supported with scientific evidence”. Should the second instance of evidence be changed to “consensus” in line with the arguments in this portion?

***Author’s Reply*:**

I agree fully with your suggestion. Your suggested rewording is far more precise and aligned with the actual data:

“People were more likely to support the NNPs not only if they believed there is scientific evidence on issues that are actually supported with scientific evidence (e.g., efficacy of vaccines to prevent severe illnesses or deaths), but also on issues where the scientific consensus is not available, such as wearing masks while driving alone.”

9) In pg. 34, the first and second paragraphs need to be written a bit more clearly. In the first para, the sentence starting with “People were more likely…”, is the suggestion that people believe in scientific evidence even in some instances where scientific consensus is not available? In the second para, am I right that in line 5 (sentence starting with “However...”), risk needs to be qualified as “perceived risk”? Also, perhaps change to “lay people have the power” from “to power”?

***Author’s Reply*:**

Thank you for encouraging me to be far more precise. I have now revised those two paragraphs to incorporate your changes, and to provide new information about this important variable. Please see below:

“**Perceived scientific consensus**. Perceived scientific consensus pertaining to Covid-19 risks and its relationship with NNP support warrants special attention, due to its unexpectedly strong and robust impact in Samples A-D, which I then further examined in Sample E (2022). People were more likely to support the NNPs not only if they believed there is scientific evidence on issues that are actually supported with scientific evidence (e.g., efficacy of vaccines to prevent severe illnesses or deaths), but also on issues where scientific consensus is not available, such as wearing masks while driving alone. Participants’ responses were so consistent that all items – supported and unsupported – yielded a single, interpretable assessment. Despite this being a supplementary indicator (and thus was given to half of 1,200+ participants in 2021-based samples), it unexpectedly emerged as the single strongest predictor of NNPs over and above control variables and core indicators.

Sample E replicated these patterns with updated PSE items, and showed that people who support NNPs believe that there is evidence behind statements such as N95 masks are safe for children’s use during a typical school day, if US had high (95%) vaccination rates, Covid-19 would have stopped spreading, and needing to prioritize children under the age of 12 for booster shots, even if there is no evidence behind those claims or WHO recommends otherwise (Table 6). These results suggest that it is possible that the PSE items did not adequately measure participants’ perceptions of risk or risk mitigation, but are more related to beliefs in scientific evidence, which is a different research questions I am currently pursuing.”

10) The last, and only major point to made pertains to the discussion around the role public perceptions of scientific consensus. There are three comments in relation to this. First, the aspect of scientific consensus arrives a bit unexpectedly in the methods without being incorporated sufficiently into the background. This would be helpful to do by way of a setting a stage for this variable especially as it seems to form an integral part of your discussion section. Second, while the author is rightfully concerned about ‘false consensus’, it would be worthwhile to bear in mind that this kind of ‘false consensus’ might have been arrived at over time after repeated exposure to excessive amounts of media coverage, interpersonal communication with trusted social networks, personal experience with COVID-19 and other engagements with the informational environment. As such, it would be useful to qualify the concern expressed by the author by contextualising it against the informational environment (or, more specifically, the infodemic), lest it might seem like the ‘false consensus’ is formed in a vacuum. Third, while it is appreciated that the discussion does not overstep its remit, it would nonetheless be useful to include a brief discussion practical implications of these findings for risk communication practice and policy.

***Author’s Reply*:**

Thank you so much once again. Indeed, there is a body of research showing that media outlets in many countries focus on predominantly negative aspects of Covid-19, where they report the worst (deaths, cases, hospitalizations), but they omit some trends that might reduce people’s fears (e.g., deaths, cases, and hospitalizations by age, relatively low risk of Covid-19 to children, and others).

I now cite those findings more clearly and note briefly what such perceived consensus might mean for our discourse (please see my answer to your previous comment). As I now note in this submission, I considered the possibility that even though my items were about risks, what I really measured (inadvertently) was participants’ beliefs. However, I found this perception of the scientific evidence variable to be so interesting and its effect so surprising that it led me to conduct a new set of studies which are now under review in a different manuscript.

Once again, thank you so much for your thoughtful feedback and support.

**REVIEWER #3 INPUT**

I would consider changing the title to make it immediately clear that the paper focuses on individuals’ own estimation of COVID-19 risks. The phrase “COVID-19 risk estimation” is a little ambiguous; perhaps something like “individuals’ COVID-19 risk perceptions” would be clearer.

***Author’s Reply*:**

Excellent idea. Upon revisiting the manuscript, I agree that being precise in the title matters greatly. I was inspired by your suggestions, so I now titled the manuscript as: “Covid-19 Risk Perceptions, Over-estimations Predict Support for the ‘New Normal’ and Continuing Restrictions Past Vaccinations”.

• The paper has several typos and grammatical mistakes. More editing is needed.

***Author’s Reply*:**

Agreed. I now edited this manuscript carefully and have solicited assistance to avoid sloppy errors and ensure accurate wording, punctuation, and grammar.

• Did you collect information about which countries participants in the Prolific international sample lived in? This information would be useful in considering how responses from those samples compare to responses from the other samples.

***Author’s Reply*:**

Yes, that information is available in my data. I decided against making it a central part of my work due to rather small sample sizes of numerous countries. The analysis that you recommend is very much necessary though it is a bit beyond the data that I currently have. My overall objective was rather modest: Test whether higher risk perceptions (over-estimations) are associated with endorsement of strict practices, and examine whether this association emerges in different samples.

I now clarify more precisely this limitation:

“Second, despite relying on participants from several different countries, the study did not examine the situational, cultural, or political factors that may explain the difference between, for instance, Australia/NZ (Sample D) and US (Sample A). Sample differences are reported in SI file. Therefore, the converging results across those samples should only be used as evidence of generalizability; they should not be used to make inferences about cross-cultural comparisons or population-level attitudes. Follow-up research would benefit from documenting empirically the social conditions, government responses, and perceptions of media that lead to differences between country-level scores. Importantly, future research should also go beyond the cross-sectional data collection and examine whether risk estimation leads to new normal endorsement (or even vice versa) over time.”

• Were the other attention check measures similar to the sample item included in the paper (“For quality control, please select ‘3’”). If so, the substantial proportion of people who failed the attention check measures is concerning, particularly in Sample C (15% of participants in Sample C failed). Can the author comment on this?

***Author’s Reply*:**

The reason for different recruited versus retained *N*s is due to my employing the pre-screening question. For instance, in some cases, I employed an initial pre-screening item that participants saw before they even started the study. An example is: "How often do you go to the movies"? The participants were instructed to ignore the question and to select both 'never' and 'once per month'. Participants who failed this question would then be directed out of the study immediately, thus increasing the quality of the participant base.

However, I learned that this is not the desired strategy, and although I specified it in my informed consent, some participants responded negatively to this tactic. Whether this tactic is appropriate for researchers to use or not requires a broader conversation. My experience with online platforms has been that a large majority of workers are conscientious.

I admit that I am not sure whether this adequately explains the differences between recruited and retained samples. To remedy this challenge further, I note in the manuscript that the results and conclusions remain substantively unchanged when analysing the data using all recruited responses and when only using participants who passed the rigorous attention checks. With the hope of encouraging future research, I also make my data available to others.

• One of the items capturing support for new normal practices in Table 3 is “Implement a program similar to COVID-19.” Is this item missing a word or does it contain a typo? I don’t understand what it means.

***Author’s Reply*:**

Thank you for bringing this to my attention. You are right that this is an error; the copy/pasted image accidentally cut off the complete wording. I now took care to present the complete image. The missing statement reads: “Implement a program similar to Covid-19 to tackle flu, which kills thousands every year.”

• What does the shading of certain rows in Table 5 and Table 6 indicate? This should be explained in the table notes.

***Author’s Reply*:**

Thank you for bringing this to my attention. I have now made extensive revisions to my tables and I have provided clear explanations for different items. There is no more shading.

• Can the author comment on why they chose not to make a scale using the core risk-estimation indicators and instead included them as separate predictors in the regression model? Given that the items are correlated with one another, including them all in the model may have resulted in variance inflation. Additionally, given the number of correlated variables included as predictors in the model, you should test the joint significance of the core risk-estimation indicators rather than looking at the individual correlation coefficients.

***Author’s Reply*:**

Thank you for this excellent suggestion. Yes; I have followed your advice and I have now made extensive changes to the way I present my regression results. I do not reproduce all text due to its length. In summary, I now write the following (Page 33):

“Next, I conducted regression analyses to examine the relationship between core Covid-19 indicators and NNP support considering the interrelated nature of multiple indicators. Specifically, I: 1) assessed the effects of core indicators on NNP, 2) examined this relationship while considering the potential controls, and 3) assessed the predictive power of perceived scientific consensus on NNP over and above all other variables. Because of the high correlation between three core indicators (estimate the chances of a health person dying, ending up in ICU, or never recovering; *r* > .70, *p* < .001), I averaged them into a single estimate to reduce multicollinearity.”

• There is a sentence fragment on page 24 of the manuscript: “However, what makes the label ‘over-estimation’ a more appropriate descriptor as opposed to ‘higher estimation’.”

***Author’s Reply*:**

Thank you for bringing this to my attention. I fixed this statement as follows (underlined component reflects the change):

“However, what makes the label ‘over-estimation’ a more appropriate descriptor as opposed to ‘higher estimation’ requires an assessment of participants’ own responses and the currently known evidence.”

The next paragraph proceeds to address this question and remains unchanged.

• In general, I think the author may just be trying to fit too much into one paper. With so many different variables and analyses to describe, I found the paper difficult to follow. I think tightening the organization of the paper and being as concise as possible could help with this. It would also be helpful to lay out the paper’s main hypotheses more explicitly are and describe the analyses used to test each one.

***Author’s Reply*:**

I happen to fully agree with you. Revisiting my manuscript after a few months have passed was illuminating, educational, and encouraged me to make major revisions. In this current version, I ensured that I clearly state that I only wish to examine one relationship: Risk (over)-estimation and support for the new normal. However, I test that one relationship in variety of ways with hope of extending the generalizability of my findings. Specifically, I note the following (transition section titled “Present Study” placed before *Methods*):

“In this study I tested the possibility that people will likely over-estimate Covid-19 risk, and that their over-estimations would be associated with stronger support for continuing restrictions even after the most vulnerable populations have been vaccinated and after the threat of overwhelmed hospitals has abated. My expectations that people would over-estimate Covid-19 risks are based on scholarly evidence that media focuses a negative- and fear-based approach to Covid-19 (43, 44), and on representative sample polling showing that people over-estimate risks (16). My expectation that these over-estimates would be related to endorsement of the new normal is based on literature of risk-estimation, availability heuristics, and moralization summarized above.

I test a single relationship in this study: risk (anticipated over-)estimation and support for the new normal. I sought to increase confidence in my findings by drawing from different samples and by measuring laypeople’s support for the new normal and risk perceptions through multiple complementary indicators. In addition, I examine other variables that may inform future research on this topic (political ideology, belief in conspiracy theories that devalue Covid-19, general concern over Covid-19, and general compliance with restrictions).”

Finally, I have also separated my analyses into the main section (Part I reporting the results using the core indicators) and the alternative indicator section (Part II reporting the knowledge-based results). I also simplified my presentation by shifting some non-central exploratory variables to my Supplementary Online Materials. In doing so, I wanted to remain transparent yet succinct.

• In my view, the major limitation of this paper is its cross-sectional nature. Using this data, it is not possible to say whether over-estimation of COVID risks leads to ‘new normal’ policy support or vice versa. The author should discuss the possibility of reverse causation and its implications in the paper.

***Author’s Reply*:**

Once again, I am in full agreement with you; that is indeed a limitation of the paper and I hope to pursue this line of research in the future. For instance, it is possible that informing participants that people tend to over-estimate certain Covid-19 risks reduces their support for the ‘new normal’ policies (of course, that might also have some unintended consequences of reducing compliance).

To temper the claims in this manuscript, I now ensure that I avoid language that infers any causality. In addition, I now provide a more thorough explanation of this limitation in the general discussion:

“Importantly, future research should also go beyond the cross-sectional data collection and examine whether risk estimation leads to new normal endorsement (or even vice versa) over time.”

I also identify the cross-sectional nature of my data as a limitation in regards to the concern variable (Pages 48-49)”

“Fifth, I assessed participants’ concern over contracting Covid-19 in all samples. However, I inadequately explored its role because it is not obvious whether its relationship is one of a predictor or a dependent variable. To be sure, that challenge is present in my other interpretations due to cross-sectional nature of this data. From a theoretical perspective, however, it is plausible that concern over contracting Covid-19 leads one to selectively consume fear-based information, causing them to over-inflate the risks; or, it is equally plausible that over-inflation of risks cause one to be more concerned. Path analyses support both speculations (available from author). Because the nature of the two variables is likely mutually reinforcing (135), future research can acknowledge that concern over Covid-19 has value and consider how it relates to risk-estimation and NNP endorsement over time.”

Once again, thank you so much for providing such thoughtful comments and for encouraging me to make my work more defensible. I am confident that thanks to your and other reviewers’ input, this manuscript is greatly improved.
