# Supplementary material for: The new normal: Covid-19 risk perceptions and support for continuing restrictions past vaccinations
Source: PLoS One. 2022 Apr 8;17(4):e0266602. doi: 10.1371/journal.pone.0266602 (PMC8993013; doi:10.1371/journal.pone.0266602)
Supplement: Attachment — Submitted filename: PLOSONE responses.docx [file pone.0266602.s013.docx]

**Dr. Hanna Landenmark**

*Senior Editor PLOS ONE*

**Santosh Vijaykumar**

*Academic Editor PLOS ONE*

**PONE-D-21-17141R1**

**Covid-19 Risk Perceptions, Over-estimations Predict Support for the ‘New Normal’ and Continuing Restrictions Past Vaccinations**

Thank you so much for inviting me to resubmit my manuscript once again. I was delighted to be given a chance to revise and strengthen my work. As requested, I made the following changes to my work.

**In summary, I made the following changes:**

1. **Throughout the manuscript, please replace Covid-19 with COVID-19 (all caps)**
   a. Done. I made all requested changes to COVID-19 term.
2. **Introduction, pg. 4, amend (i.e., deaths of Covid-19 or hospitalizations) to (i.e., reduction in deaths of Covid-19 or hospitalizations).**
   a. Done. I made the following adjustment:

“Whether to continue restrictions is not a decision that can be made exclusively by health scientists because societal well-being cannot be reduced to a single indicator of success (i.e., reduction in deaths of COVID-19 or hospitalizations); instead, it involves consideration of competing priorities and limited resources (mental health, economics, and education, among others) (11, 12).

3. **Introduction, pg. 4, 3rd para, 3rd line: replace "underestimation of COVID-19" with "under-estimation of the threat of COVID-19" (or any other qualification you feel might be more appropriate).**
   a. Done. I made the following adjustment:

“For instance, under-estimation of the threat of COVID-19 or belief in conspiracy theories (e.g., unfounded cures) may lead people to disregard health-minded rules and risk the lives of the vulnerable (13-15).”

4. **Pg. 5, end of first para, please qualify the term "informational and reputational cascades" and the "moralization of COVID-19".**

Thank you for encouraging me to be more precise. I did not qualify those statements in this statement, only because I do so in the subsequent section “Origins and Catalysts of Risk Over-estimation: Theoretical Foundation”. I have now refined the definitions and state the following:

“I proceed to explain how my predictions are informed by complementary theories on perceptions of unknown (vs. known) risks (18), availability and reputational cascades (4, 5, 17, 18), and moralization of COVID-19 (19, 20).”

The following section defines these terms:

“While erring on the side of extreme caution is defensible in the absence of information, continuing to do so in light of new information is not. This fear-based focus can further perpetuate availability cascades where new information is not used to revisit the cost-benefit analysis but is selectively disseminated and censored; information that deviates from the narrative may elicit reputational damages or moral outrage (20, 21, 28). These forces may also be explained with moralization, a process by which an attitude becomes a matter of moral imperative (20). When an attitude becomes moralized, it becomes absolute, intolerant, and resistant to change, which may further perpetuate the availability cascade of moralized information.”

5. **In Table 4, for #1 it is not clear what the estimate should be, as it has been provided for the other statements/questions.**
   a. Thank you. May I clarify that you are referring to the Core Indicator 1 (What is the average age of a person who died of COVID-19 in the US?)? If so, the estimation benchmark is 78+ (noted in Table 4):

“The average age for deceased COVID-19 patients tends to be approximately 80 years of age, with some variation based on gender and reporting protocols (36, 45-51).”

6. **In Table 5, the word 'consensus' is misspelt in items #1 and #3.**
   a. Done. Thank you for bringing this error to my attention. I edited my manuscript one more time and detected a few similar errors in ‘consensus’.
7. **In pg. 21, 2nd para, line 6, there's a phrase: "aggregating items that less empirical support" which needs correction.**
   a. Thank you for bringing this error to my attention. The statement lacked ‘that’. Accordingly, I changed it to: “I considered evaluating the data using a 5-item scale aggregating items that have less empirical support (PSE –supported; α = .78), and a 5-item scale aggregating items that have more empirical support (PSE – unsupported; α = .86).”
8. **In pg. 28, it'd be useful to provide a brief description of the "lab leak hypothesis" for the benefit of the readers who might not be familiar with this issue.**
   a. Done. I now changed the description to the following: Since the data collection (April, 2021) and while the manuscript was under review, the so-called “lab leak hypothesis” (i.e., belief that COVID-19 emerged from a lab) has received more attention and according to Maxmen and Mallapaty (106), many research institutes are calling for a deeper investigation of this possibility.
9. **In pg. 31, the phrase: "SI provides additional data based 2021 samples" needs to changed to "...based ON 2021 samples"**
   a. Done. The line now states “SI provides additional data based on 2021 samples, including a visual representation of frequency distributions, which can aid readers in evaluating this work.”

**Besides these changes, I also made the following minor adjustments:**

1. I reported median estimation scores, in addition to mean and standard deviation.
2. I replaced the image-based tables with Word-based tables. The actual numbers remained the same, but the Word file is cleaner.
3. I edited the manuscript for clarity and precision.

Thank you once again for your continuing support. I look forward to your feedback.

~ Maja Graso

University of Otago

New Zealand
